# Advances in Multiple Sclerosis

**DOI:** 10.3390/biomedicines13020266

**Published:** 2025-01-22

**Authors:** Victor M. Rivera

**Affiliations:** Department of Neurology, Baylor College of Medicine, One Baylor Plaza, Houston, TX 77030, USA; vrivera@bcm.edu; Tel.: +1-832-407-0668

## 1. Introduction

Multiple sclerosis (MS) is the most common demyelinating and inflammatory disease of the CNS; it has a global distribution with increasing prevalence [1]. MS affects individuals of most ethnicities [2], placing a substantial regional socioeconomic burden in a vast range of geographic areas [3]. This disease poses numerous challenges for individuals, including the need to cope with the still unidentified aspects involved in its mechanistic processes, including a multifactorial etiological complex influenced by environmental epigenetic factors [4], as well as the immune and inflammatory molecular mechanisms involved in the development of the disease [5]. With the evolution of knowledge in this field, some of the elements discovered may eventually be helpful as diagnostic markers, and some have the potential to be translated into therapeutic targets. In recent decades, studies on MS have been carried out in many branches of neuroscience. Epidemiologically, this disease is observed in the highest frequency in the northern- and southern-most areas of the world: Canada, US, Scandinavia, the British Islands, most of Europe, New Zealand and Australia. An inheritable genetic factor is recognized to comprise at least one-third of the complex MS etiological make-up, identified as the HLA-DRB1 * 15:01 [6]. The current theory explaining the world-wide dissemination of this disease, including the Americas, the Middle and Far East, is the European genetic risk factor, passed on throughout history though military invasions, migration and other historical and socio-political phenomena [7,8]. While the causality of MS is multifactorial, autoimmunity is the main driver behind its mechanisms [9]; it is involved in the initiation of the characteristic, erroneous, autoimmune cascade, a response of the innate immune system to an antigen, followed by the participation of the adaptive immune system. The latter phase is essentially responsible for the structural damage recognized in the disease, inflammation, demyelination and neurodegeneration, caused by cellular and humoral factors. Pathological alterations are not restricted to white matter but are also present in the gray matter of the CNS, including abnormalities in synapses and synaptic networks. These abnormalities have also been observed in animal models of MS in research laboratories. A critical molecular step in the pathogenesis of this disorder is the traffic of activated immune cells through the blood–brain barrier (BBB), particularly their deleterious effect on the CNS. Some of the aforementioned elements of the disease have been discussed in previous works [10]. The neurological clinical manifestations are extremely varied, establishing MS as one of the most symptomatic diseases in human pathology. The current clinical classification considers several dynamic varieties conditioned by the activity of the disease, whether this manifests clinically (exacerbation, acute relapse, increased EDSS) or as MR imaging behavior (T1 lesions enhancing post-infusion of contrast material, appearance of a new T2 or enlargement of a previous T2 lesion). The identified clinical varieties, i.e., relapsing/remitting disease, and secondary progressive or primary progressive MS, may transform into a worse form of the disease, subject to factors associated with its activity [11]. The nomenclature of the MS clinical classification continues to evolve. At present, the term “Clinically Isolated Syndrome” generally refers to the first event, or the initiation of the relapsing/remitting form of the disease (if the rest of the diagnostic criteria are met). New concepts such as Progression Independent of Relapse (PIRA) and Relapse-Associated Worsening (RAW) are recognized in clinical situations, and are most likely to be incorporated in future classificatory proposals. Undetected inflammation, a chronic low-level inflammatory process occurring in the CNS, is identified as the underlying pathology of PIRA, most likely being reflected clinically as a form of secondary progressive MS [12]. The diagnosis of MS becomes more challenging as our understanding of this disease increases in complexity. To assist in diagnostic accuracy and prevent misdiagnosis, diverse diagnostic criteria have been established. In the modern era, different versions of the so-called McDonald Criteria have been produced by different international panels, starting in 2001. Each version has incorporated updated knowledge on this condition through MRI findings, as well as laboratory and clinical endeavors. The most recent version of the McDonald Criteria, created in 2017 [13], will be replaced by the 2024 version, which is to be officially published and implemented in 2025. The development of therapeutic molecules for MS over the last three decades has exceeded that of available treatments for any other neurological disease. All approved MS therapies have demonstrated significant effects in controlled trials, including reductions in annualized relapse rates and in the progression of the disease, as well as improvements in MRI activity. The large variety of therapies, including interferons, glatiramer acetate, teriflunomide, fumarates, S1P1 receptor modulators, cladribine, and the monoclonal antibodies antiɑ4-integrin, antiCD20 and antiCD52, have been classified as being of high, moderate or modest efficacy, adhering to the guidelines provided by the Association of British Neurologists (ABN 2015 guidelines), according to their average relapse reduction [14]. This classification helps practitioners in selecting the appropriate therapy when considering the degree of clinical and MRI aggressiveness presented by the case.

**An Overview of Published Articles**. This Special Issue addresses *Advances in Multiple Sclerosis*, celebrating the 10th Anniversary of *Biomedicines*, and consists of nine articles covering a broad range of themes that will be briefly discussed in the following paragraphs. The purpose of this Special Issue is to highlight these studies and encourage readers to engage with this ever-relevant topic. El-Samad et al. [15] studied the role of glutamate excitotoxicity in MS pathogenesis, particularly early changes at the photoreceptor synapses, related to the visual system. Using quantitative and qualitative immunofluorescence microscopy and Western blotting, this study analyzed whether the EAAT5 glutamate transporter could be involved in the structure alterations and function of glutamatergic photoreceptor ribbon synapses in an EAE mouse model of MS. The data showed that EAAT5 was strongly reduced at the photoreceptor synapses of EAE retinas in comparison with photoreceptor synapses of the control retinas, and Western blot analyses demonstrated decreased EAAT5 expression in EAE retinas. The authors encourage future investigations to be conducted, in order to further elucidate the precise mechanisms involved. Coagulation components have been shown to provide immunomodulatory and proinflammatory effects in the CNS, resulting in inflammation and degeneration. Whether patients with MS exhibit an overrepresentation of polymorphisms implicated in coagulation and if these genes are associated with disease progression and disability were studied by Hadjiagapiou et al. [16]. The cardiovascular disease (CVD) strip assay performed, analyzing 11 genetic polymorphisms associated with thrombosis and CVD in MS patients and controls, showed that PAI-1 5G/5G homozygosity was more frequently observed in MS patients (OR:6.33 (95% CI: 1.32–30.24); *p* = 0.016). Advanced neurological disability (*p* = 0.03) and disease worsening (*p* = 0.02) were demonstrated in carriers of the HPA-1a/1b polymorphism. These findings suggest that MS may be linked to thrombophilia-related polymorphisms, hence warranting further investigation. The review article by Kee et al. [17] discusses the role of meningeal tertiary lymphoid structures (TLS) or B-cell follicles, and compartmentalized inflammation (trapped inflammatory cells) in the form of organized clusters of immune cells in the connective tissue spaces of the vasculature and leptomeninges behind an intact BBB. These pathophysiological processes are associated with the most severe clinical forms of MS: PIRA and primary progressive MS. The contributors to this review feel that TLS could represent an important marker of disease activity, as well as a therapeutic target for mitigating the most severe outcomes of disease. Huss et al. [18] performed a prospective study investigating the composition of the gut microbiota in MS patients, the presence of the *Clostridium perfrigens* epsilon toxin in serum, and the influence of some disease-modifying therapies (DMT) on epsilon toxin levels and on microbiota. The MS cohort in this study comprised patients with relapsing/remitting disease treated with the therapeutic molecules teriflunomide and fingolimod, and the monoclonal antibodies natalizumab and ocrelizumab. Untreated patients were also included. Neither epsilon toxin nor antibodies against it were detected. No significant differences in the relative abundance of fecal microbiota in the gut microbiota of MS patients receiving DMT were observed. This study did not show evidence supporting the hypothesis of a causal relationship between the *Clostridium perfringens* epsilon toxin and MS. Neurogenic lower urinary tract dysfunction (NLUTD) is common among MS patients regardless of the clinical type and duration of disease. While uroflowmetry (UF) is performed by some authors instead of urodynamics (UD), the inter-rater reliability concerning diagnosis and therapy based on UF remains challenging. Jaekel et al. [19] prospectively studied 92 people with MS (PwMS), classified using the diagnostic criteria of ‘normal findings’, ‘detrusor overactivity’, ‘detrusor underactivity’, ‘detrusor -sphincter dyssynergia”, and ‘bladder outlet obstruction’. Four raters assessed the diagnostic criteria, which were applied to a possible treatment scheme that included ‘no treatment’, ‘catheter placement’, ‘alpha-blockers’, ‘detrusor-attenuating medication’, botulinum toxin’, neuromodulation’, and ‘physiotherapy/biofeedback’. The authors noted the high influence of the individual raters (kappa rating for diagnosis = normal findings; kappa rating for therapy = botulinum toxin), and that UD remains the gold standard for the diagnosis of NLUTD in PwMS. Reverchon et al. [20] utilized three MS groups, acute relapsing patients (ARMS), natalizumab-treated (NTZ), and control subjects, to study the serotonin receptor 5-HT7 expressed on circulating lymphocytes and evaluated the effects of its activation on cytokine production with peripheral blood mononuclear cell (PBMC) cultures. The researchers found a significant increase in the 5-HT7 surface expression on T lymphocytes and on the different CD4 + T cell subsets exclusively in NTZ-treated patients. These findings strengthen the evidence that 5-HT7 may play a role in the immune-protective mechanisms of NTZ in MS disease. This work constitutes the first study on the receptor 5-HT7activating naïve T cells and influencing the inflammatory response in MS persons treated with NTZ, a humanized monoclonal antibody against the cell adhesion molecule ɑ4-integrin. In the pathologic MS microenvironment, ɑ4-integrin facilitates the traffic of immune cells from the peripheral circulation into the CNS. NTZ inhibits this process. The research study by Geβner et al. [21] demonstrated the importance of detecting subtle motor deficits in people with MS below the clinical threshold, in the early stages of the disease, utilizing complex motor functions. These methods have potential use as baselines and as assessments throughout the course of the disease. The study evaluated the countermovement jump (CMJ), a high-challenge task in the form of a maximal vertical bipedal jump (three maximal CMJs) on a force plate, in 99 PwMS and 33 healthy controls (HCs). These cohorts did not differ significantly in age, disease duration, body mass index (BMI) or EDSS. PwMS demonstrated significantly decreased CMJ performance in terms of kinetic, temporal and performance parameters (*p* < 0.05). The authors encourage longitudinal studies to be performed to evaluate whether the CMJ can be used to measure disease progression. In another study [22] by Geβner et al., the authors expanded their evaluations of the CMJ in association with age, sex and BMI, utilizing 164 PwMS and 98 HCs, within a cross-sectional design. All participants performed three maximal CMJs on a force plate. Analysis of the dynamic components of the CMJ showed the most significant effects of sex on flight time (η^2^ = 0.23), jump height (η^2^ = 0.23), and positive power (η^2^ = 0.13). PwMS showed significantly lower CMJ performance compared to HCs in middle-aged (31–49-year-old) individuals, with normal weight to overweight and in both women and men. The researchers concluded that age, sex, and BMI are associated with muscle mechanical function in PwMS and HCs, and emphasize the value of integrating the CMJ into the diagnostic assessment of people with early MS and in developing individualized neurorehabilitative therapy. The article by Moore et al. [23], investigating the MS healthcare disparities among ethnic minorities, including Hispanic and Black populations in the United States, addresses a sociological concern shared by those in countries with a high prevalence of the disease: increasing frequencies of MS among minorities, and by extension, increasing frequencies affecting new migrants as well. This review emphasizes the current understanding about the patterns of disease presentation, genetic considerations, response to treatment, and future directions of inquiry, subsequently proposing practical methods with which to overcome these challenges.

## 2. Conclusions

This Special Issue, Advances in Multiple Sclerosis, provides the reader with a varied collection of articles conveying very recent discoveries related to genetic markers and the contribution of coagulation factors to the multifactorial etiology of MS; the lack of confirmation of a relationship between the *Clostridium perfringens* epsilon toxin and this disease is also highlighted. The utilization of uroflowmetry in the evaluation of neurogenic lower tract dysfunction (a rather common problem in people with MS regardless of age or clinical type), and the challenges posed by interrater variability, are discussed in a well-designed study addressing these questions. Tertiary Lymphoid Structures constitute the meningeal sites of compartmental inflammation in the CNS, and the pathophysiological path for progressive forms of MS. An original study demonstrated the T-lymphocyte receptor 5-HT7 may play a role in the immune-protective mechanism of the high-efficacy monoclonal antibody natalizumab. One study reports that the CMJ detects subtle motor deficits below the clinical threshold in MS, and another paper from the same laboratory describes neuromuscular and muscle mechanical function, as assessed by the CMJ, associated with age, sex and BMI. Concerns about the health disparities affecting minorities, particularly Hispanic and Black populations in the US, are studied, with future directions being suggested by another group of authors. Thus, an invaluable, comprehensive review of these findings is presented in this Special Issue.
